# Poorly differentiated carcinoma with neuroendocrine features in two patients with sinonasal tumours: a report of two cases

**DOI:** 10.11604/pamj.2022.42.37.34513

**Published:** 2022-05-16

**Authors:** Victor Chimezie Okebalama, John Ifeanyi Nwadiokwu, Ehioghae Osaze, Ayeni Akinwunmi Ayodeji, Obinna Mike Chinatu-Nwankwo

**Affiliations:** 1 Department of Histopathology, Babcock University Teaching Hospital, Ilishan-Remo, Ogun State, Nigeria,; 2 Benjamin Carson (Snr.) College of Health and Medical Sciences, Babcock University, Ilishan-Remo, Ogun State, Nigeria,; 3 Department of Surgery, Babcock University Teaching Hospital, Ilishan-Remo, Ogun State, Nigeria,; 4 Department of Internal Medicine, Federal Medical Center, Umuahia, Abia State, Nigeria

**Keywords:** Poorly differentiated, neuroendocrine, sinonasal, tumour, case report

## Abstract

Neuroendocrine tumors can be described as rare, heterogeneous, epithelial tumours with principally neuroendocrine differentiation that can be harboured in the sinonasal cavities. Owing to the intermingling of its clinical, radiological and histopathological features, the diagnosis of poorly differentiated sinonasal carcinomas with neuroendocrine features is a daunting one. Many of these tumours have a poor prognosis. In our two cases, patients presented with nasal cavity obstruction and growth of 8 & 5-months duration, respectively. Intranasal growths with intracranial extensions were noted in computed tomography scans in both patients. Our first patient failed to complete his cycle of induction chemotherapy (Cisplatin and 5-fluorouracil) and died 8 months after presentation while our second patient completed his cycle of induction chemotherapy but died 1year, 6months, after presentation, as he was unable to get the planned radiotherapy. Indeed, late presentations, intracranial metastasis, and poor treatment compliance can contribute to the poor outcome of these tumours.

## Introduction

Sinonasal malignancies are uncommon, representing only 1% of all neoplasms [1]. They mostly originate from the nasal sinuses and regions of the base of the skull. It represents just about 3% of the entire neoplasm of the aerodigestive tract [2]. Sinonasal malignancies with neuroendocrine features are rare head and neck tumors many of which have a poor prognosis. This is compounded by the diagnostic challenge of distinguishing these carcinomas, as it determines patient management and prognosis. These tumours can be divided into three major histological phenotypes; olfactory neuroblastoma (ONB, formerly known as esthesioneuroblastoma), sinonasal undifferentiated carcinoma (SNUC), and sinonasal neuroendocrine carcinoma (SNEC) [3]. Neuroendocrine neoplasms are classified into well-differentiated (typical carcinoids), moderately differentiated (atypical carcinoids), and poorly differentiated (small and non-small cell types). The poorly differentiated neuroendocrine carcinomas are very rare and aggressively malignant with a high recurrence rate and the ability to metastasize to other sites through blood and lymphatic channels [4, 5]. We herein discuss two cases of this rare neoplasm to increase awareness among clinicians as well as patients. Furthermore, we will discuss clinical, radiological, histological, and immunohistochemistry findings of this tumour.

## Patient and observation

### Clinical case 1

**Patient information:** a 45-year-old male presented to our surgical outpatient unit in February 2021 with right nasal cavity obstruction over the preceding 8 months and ulcerated swelling on the nasal bridge that progressively enlarged over a duration of 3 months. He also complained of associated progressive proptosis and reddening of the right eye, anosmia, recurrent severe headache, declining right eye vision, significant weight loss, irrational behavior, and restlessness. The medical history was not contributory. His family and psychosocial history revealed that he was married in a monogamous family setting. The wife is 38-year-old. They both have a tertiary level of education. He does not smoke or drink. There was no history of the use of recreational drugs. His genetic history showed no family history of previous malignancy. Symptoms had persisted despite the use of several medications of unknown names over the preceding 8 months prior to presentation.

**Clinical findings:** on clinical examination, an ulcerated, irregularly shaped mass on the face, partially obliterating the right nasal cavity, extending from the nasal bridge to approximately 3 cm above the glabella was seen. The mass was firm in consistency with a punctum discharging serous effluent.

**Figure 1:**
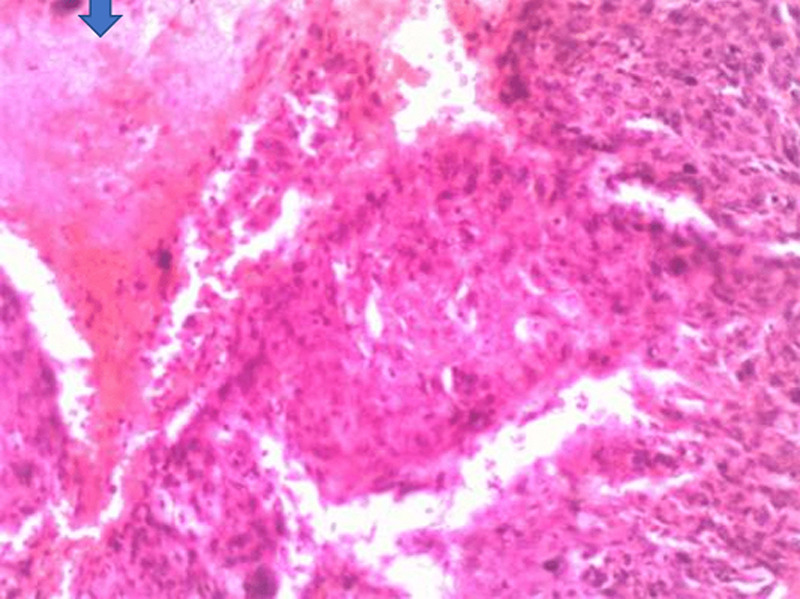
(H & E x 100); sections show sheets and nests of pleomorphic cells having mainly hyperchromatic nuclei with mild to moderate eosinophilic cytoplasm, also seen is a focal area of necrosis (arrow)

**Diagnostic assessment:** haematological investigations including full blood count (FBC), renal function test (RFT; Serum electrolytes, urea, and creatinine), and liver function test (LFT) done were all within normal ranges. Computed tomography (CT) scan showed a large soft tissue in the right nasal cavity extending into the ethmoid sinus. Left orbital and intracranial extensions were also noted. The histopathological evaluation from a collected nasal biopsy showed highly pleomorphic cells disposed in sheets and nests. The component tumour cells had mainly hyperchromatic nuclei with mild to moderate cytoplasm. Seen also were atypical mitotic figures and focal areas of necrosis with infiltration by acute and chronic inflammatory cells (Figure 1). Furthermore, immunohistochemistry revealed that the neoplastic cells were strongly positive and focally positive for cytokeratin (AE1/AE3) and Neuron-Specific Enolase (NSE), respectively. They were, however, negative for S100, Desmin, synaptophysin, and CD45. The immunohistochemistry was, however, done in another facility as our hospital was not able to offer it.

**Diagnosis:** following a histopathological investigation, an initial diagnosis of a sinonasal neuroendocrine tumour was made. However, a confirmatory diagnosis of poorly differentiated sinonasal neuroendocrine carcinoma was made following immunohistochemistry.

**Therapeutic interventions:** we opted against surgery in his case, considering the intracranial tumor involvement. Rather, chemotherapy to be followed by radiotherapy was planned for him. Following a repeated baseline investigation made up of FBC, RFT, and LFT, our patient was commenced on induction chemotherapy of a combination of Cisplatin and 5-fluorouracil 3 days after obtaining his immunohistochemistry report. The patient, however, declined to receive his complete cycle of chemotherapy despite persuasion as he received only two of the planned three cycles of the induction chemotherapy at 3 weeks intervals. On each cycle, following the aforementioned baseline investigations, he was given as follows: Pre-medication drugs: Injections Dexamethasone (8mg), Ranitidine (50mg), and Ondansetron (8mg) in 100mls of normal saline over 30 minutes. Then followed by Injection Cisplatin 100mg/sq.mt in 500mls normal saline over 2 hours on day 1. On Day 2, he received pre-medication, then injection 5-fluorouracil 750mg/sq.mt in 500mls normal saline over 20 hours.

**Follow-up and outcome of interventions:** although our patient didn´t show up for follow-up visits even as there was poor compliance to planned intervention, he, unfortunately, died 8 months following his first presentation. Furthermore, there were no adverse reactions or unanticipated events.

**Patient perspective:** in the absence of our patient, his wife wrote thus: my husband received optimal, timely, and professional treatment from the doctors at Babcock University Teaching Hospital Ilishan-Remo, Ogun State. Although he refused to receive his drugs at some point despite the doctors caring persuasion at the hospital leading to his early death, I´m sincerely grateful for all the efforts put in by his doctors at the hospital.

**Informed consent:** it was was obtained from the wife, who was his caregiver and next of kin.

### Clinical case 2

**Patient information:** a 52-year-old male presented to our surgical outpatient unit in June 2020 with complaints of left nasal growth and obstruction of 5 months and 3 weeks duration. There were also complaints of left epistasis, poor vision, and irrational behavior. He had no significant medical history. His genetic history showed that there was no family history of malignancy. Furthermore, His family and psychosocial history showed that he was married in a monogamous setting. The wife is 43-year-old and both have tertiary levels of education. There was no history of smoking, alcohol consumption, or the use of recreational drugs. Prior to his presentation, he had been to several herbalists where herbal medications of unknown names were applied and administered to him with no improvement in symptoms.

**Clinical findings:** clinical examination revealed a left nasal mass that was erythematous and extended to the nasal bridge.

**Figure 2:**
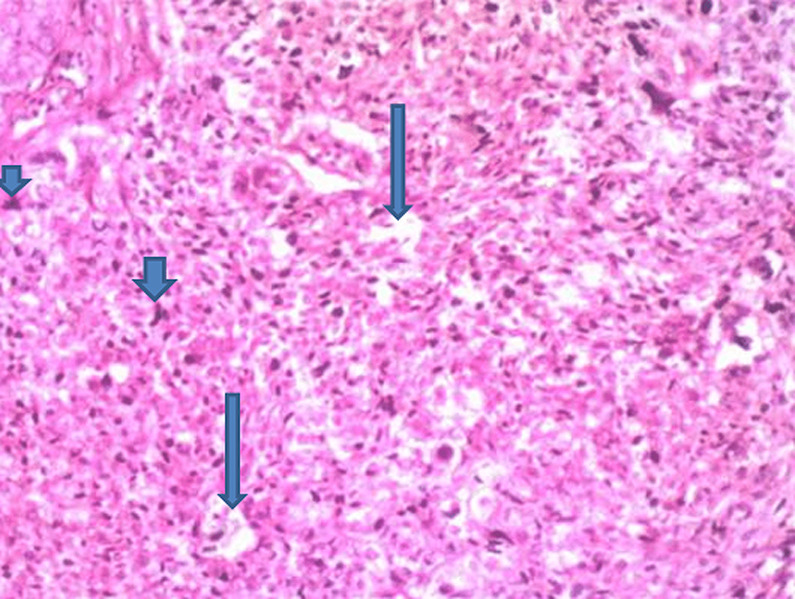
(H & E x 100); sections show sheets and vague nests of tumor cells having hyperchromatic to vesicular nuclei, indistinct nucleoli, and scant cytoplasm on a minimal fibrillary neural matrix, there are attempts at rosette formation in areas (Long arrow), also seen are atypical mitoses (short arrow)

**Diagnostic assessment:** a computed tomography (CT) scan done showed a left para-sinus soft tissue growth that was locally destructive and extended to the ethmoid sinus. There were also left orbital and intracranial extensions (infratemporal fossa). The histopathological examination of the nasal biopsy revealed atypical mitoses, sheets, and vague nests of tumour cells having hyperchromatic to vesicular nuclei, indistinct nucleoli, and scant cytoplasm on a minimal fibrillary neural matrix. There are attempts at rosette formation in areas (Figure 2) while on immunohistochemistry, the neoplastic cells showed strongly positive for both Neuron-Specific Enolase (NSE) and epithelial membrane antigen (EMA). They were negative for S100, Desmin, synaptophysin, and CD45. The immunohistochemistry was, however, done in another facility as our hospital was not able to offer the investigation.

**Diagnosis:** an initial histopathological diagnosis of a poorly differentiated sinonasal neuroendocrine tumour was made. This was further confirmed immunohistochemically.

**Therapeutic interventions:** we decided against surgery in his case, considering the intracranial tumor involvement. Rather, chemotherapy to be followed by radiotherapy was planned for him. Following a baseline investigation made up of FBC, RFT, and LFT, our patient was commenced on induction chemotherapy made up of a combination of Cisplatin and 5-fluorouracil 4 days following the availability of his immunohistochemistry result. He completed his induction chemotherapies of 3 cycles at 3 weeks intervals. On each cycle, following the above-mentioned baseline investigations, he was administered chemotherapy as follows: Pre-medication drugs: Injections Dexamethasone (8mg), Ranitidine (50mg) & Ondansetron (8mg) in 100mls of normal saline over 30 minutes. Followed by Injection Cisplatin 100mg/sq.mt in 500mls normal saline over 2 hours on day 1. Premedication, then Injection 5-fluorouracil 750mg/sq.mt in 500mls normal saline over 20 hours on day 2 and Pre-medication followed by Injection 5-fluorouracil 1000mg/sq.mt in 500mls normal saline over 20 hours on day 3. He, however, failed to obtain his planned radiotherapy.

**Follow-up and outcome of interventions:** the tumour size was reduced after the completion of induction chemotherapy. There were no adverse or unanticipated events. Although he was able to complete a full cycle of induction chemotherapy (Cisplatin and 5-fluorouracil), he was unable to get the planned radiotherapy. Unfortunately, he died 1 year, 6 months, following his first presentation.

**Patient perspective:** as obtained from his wife, who was his caregiver and next of kin: the doctors I met at Babcock University Teaching Hospital were kind and prompt enough. The facility was conducive. Most sincerely, I will recommend them to anyone, despite the demise of my husband.

**Informed consent:** it was obtained from his wife, who is his next of kin.

## Discussion

The sinonasal cavities represent an anatomical region affected by a variety of tumours with clinical, etiological, and pathological features distinct from tumors of the main head and neck cancer localizations [6]. Squamous cell carcinomas remain the most common malignant sinonasal tumors (80%) followed by adenocarcinoma. Other tumors seen here are chondrosarcoma, lymphoma, salivary gland tumors neuroendocrine tumors, and mucosal malignant melanoma [7]. The defining features of neuroendocrine neoplasms are a source of ongoing debate because some neuroendocrine markers are non-specific and can be expressed in non-neuroendocrine malignancies [3].

ONBs share some of the features seen in neuroendocrine neoplasms and are difficult to differentiate from SNECs [8]. SNUCs have overlapping morphology with poorly differentiated ONBs and SNECs; thus, a wide variety of tumours occurring primarily in this site can present with an undifferentiated or poorly differentiated morphology [9]. SNECS and SNUCS are said to arise from the respiratory epithelium of the sinonasal cavity while ONBs arise from neuroectodermal cells of the olfactory epithelium [9, 10]. SNEC was first proposed as an entity by Silva *et al*. in 1982 [11]. Neuroendocrine carcinomas (NEC) of the paranasal sinus are rare, accounting for 5% of all sinonasal malignancies [12]. Out of the 21 cases of sinonasal neuroendocrine tumors reported by Babin *et al*., 12 were male and 9 were female [13]. In a similar study, there was a higher male to female ratio with 11 males to 9 females and a median age of 49.2 years [14, 15]. Indeed, SNEC occurs slightly more commonly in males and is more prevalent in the 5^th^ and 6^th^ decades, consistent with our two cases. Till now, no specific etiologic factor has been identified [8]. However, a subset of sinonasal large cell neuroendocrine carcinoma (LCNEC) is said to be HPV-related [16]. There is no strong linkage between smoking and neuroendocrine carcinoma of the paranasal sinuses [12]. The clinical features of SNEC are non-specific and can include epistasis, nasal/facial mass, nasal obstruction, anosmia, etc. some of these symptoms are similar to what is obtainable in other sinonasal tumors. Indeed, most of these features are consistent with the findings in our cases. Generally, SNEC has a very poor prognosis [17]. A good proportion of patients present with advanced disease, just as in our cases. Extensive involvement of the orbit, skull, or brain may be seen, as was in our cases.

Olfactory neuroblastoma (ONB) was formerly known as esthesioneuroblastoma. Its origin is still being debated [18]. However, there are suggestions that ONB is derived from immature olfactory neurons [18]. It is a rare tumor with no sex predilection and affects all age groups. On CT images, it is characteristically seen with peripheral tumor cysts and specified calcifications. Its histology shows uniform lobules of small round blue cells with a neurofibrillary background. The diagnosis of ONB is established by positive staining for synaptophysin and other neuroendocrine markers combined with negative staining for keratin, muscle, melanoma, and lymphoma markers [19]. Sinonasal undifferentiated carcinoma (SNUC) is defined as undifferentiated carcinoma of the sinonasal tract without glandular or squamous features and is not otherwise classifiable [20]. It is a rare tumor that often poses a significant diagnostic challenge due to its long list of differentials. Suggestive of SNUC is strong positivity for cytokeratin with negative synaptophysin and chromogranin [3]. SNUC usually exhibits focal neuron-specific enolase positivity, unlike ONB, which stains diffusely [9]. This way, ONB can be differentiated from SNUC. Expert histopathological assessment is usually needed, as sinonasal malignancies cannot be differentiated based on clinical presentation or radiological studies alone. A distinction between poorly differentiated variants of ONBs, SNECs, or SNUCs can be difficult [3].

**Figure 3:**
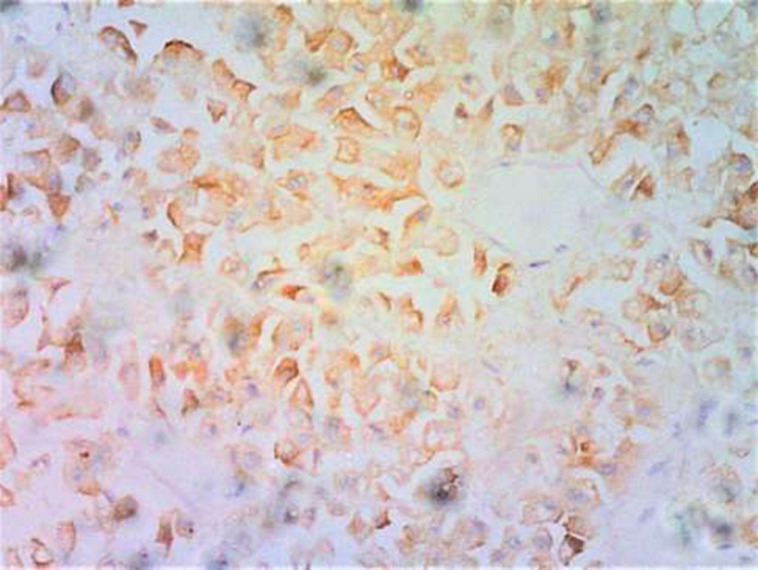
(AE1/AE3 x 100); sections show tumor cells with strong cytoplasmic staining for AE1/AE3 immunohistochemical marker

**Figure 4:**
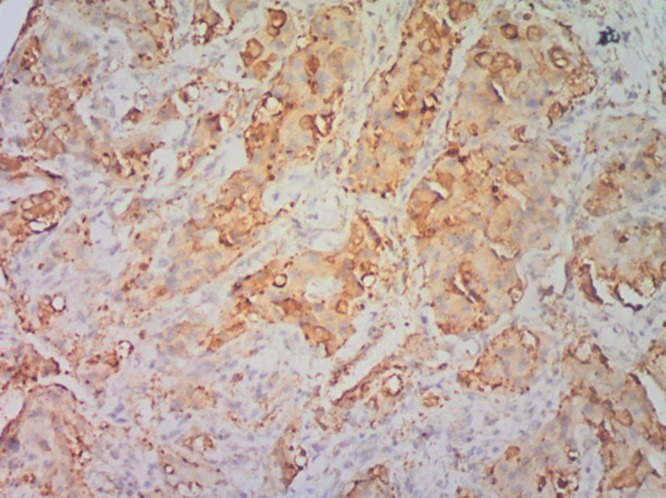
(NSE x 100); sections show moderate staining for NSE immunohistochemical marker

**Figure 5:**
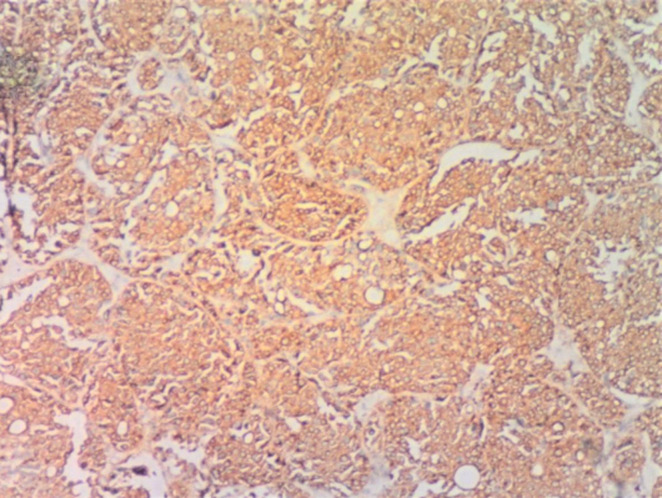
(EMA x100); sections show tumor cells with strong cytoplasmic membrane staining for EMA immunohistochemical marker

**Figure 6:**
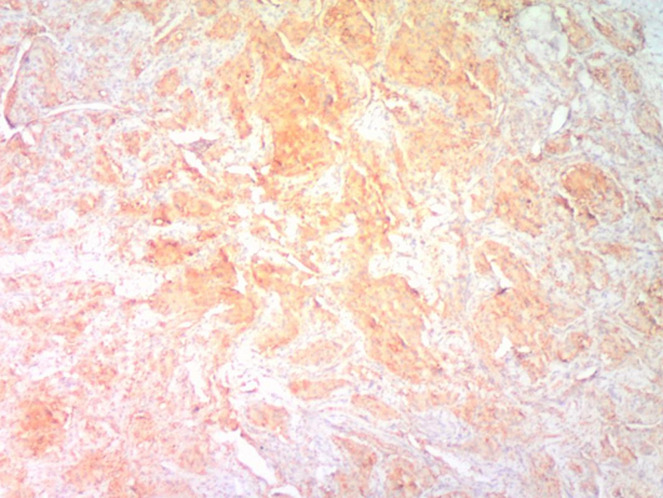
(NSE x 100); sections show moderate to strong staining for NSE immunohistochemical marker

In our first case, the histology on light microscopy showed highly pleomorphic cells disposed in sheets and nests, hyperchromatic nuclei with mild to moderate cytoplasm, atypical mitotic figures, and focal areas of necrosis with infiltration by acute and chronic inflammatory cells (Figure 1). Immunohistochemically, the tumor cells were strong and focally positive for AE1/AE3 and NSE (Figure 3, Figure 4) respectively, but negative for Desmin, CD45, and S100. In our second case, microscopically seen are sheets and vague nests of tumor cells having hyperchromatic to vesicular nuclei, indistinct nucleoli, scant cytoplasm on a minimal fibrillary neural matrix, attempts at rosette formation in areas & atypical mitoses (Figure 2). In about 80% of the tumor cells, EMA was strongly positive while NSE was positive in about 70% of them (Figure 5, Figure 6). Synaptophysin, S100, and Desmin were all negative. These features are in keeping with poorly differentiated sinonasal carcinomas with neuroendocrine features.

## Conclusion

Poorly differentiated sinonasal carcinomas with neuroendocrine features are rare but very important differential diagnoses in patients with nasal obstruction or tumor. Early presentation, greater health education, and a high clinical index of suspicion are pivotal in managing, and possibly improving the outcomes in patients with sinonasal tumors.
